# Free-Fermion Models and Two-Dimensional Ising Models Under Zero Field and Imaginary Field *i*(*π*/2)*k_B_T*

**DOI:** 10.3390/e27080799

**Published:** 2025-07-27

**Authors:** De-Zhang Li, Xin Wang, Xiao-Bao Yang

**Affiliations:** 1 Quantum Science Center of Guangdong-Hong Kong-Macao Greater Bay Area, Shenzhen 518045, China; lidezhang907@163.com; 2 Department of Physics, City University of Hong Kong, Hong Kong SAR, China; 3 City University of Hong Kong Shenzhen Research Institute, Shenzhen 518057, China; 4 Department of Physics, South China University of Technology, Guangzhou 510640, China

**Keywords:** Ising model, sixteen-vertex model, free-fermion condition, exact solution

## Abstract

The Ising model is famous in condensed matter and statistical physics. In this work we present a free-fermion formulation of the two-dimensional classical Ising models on honeycomb, triangular and Kagomé lattices. Each Ising model is studied in the cases of a zero field and of an imaginary field $i\left(\pi /2\right)k_{B}T$. We employ the decorated lattice technique, star-triangle transformation, and weak-graph expansion method to exactly map each Ising model in both cases into an eight-vertex model on the square lattice. The resulting vertex weights are shown to satisfy the free-fermion condition. In the zero-field case, each Ising model is an even free-fermion model. In the case of the imaginary field, the Ising model on the honeycomb lattice is an even free-fermion model, while the models on the triangular and Kagomé lattices are odd free-fermion models. We obtain the exact solution of the Kagomé lattice Ising model under the imaginary field $i\left(\pi /2\right)k_{B}T$, a result not previously reported in the literature. We also show that the frustrated Ising models on the triangular and Kagomé lattices in the imaginary field still exhibit a non-zero residual entropy.

## 1. Introduction

The Ising model plays a crucial role in the research of condensed matter and statistical physics of lattice systems, especially in understanding the phase transition via statistical mechanics. It has a long history starting in the 1920s [1]. The one-dimensional Ising model was first solved by Ising himself [2]. Kramers and Wannier introduced the transfer matrix method to rederive the exact solution for the one-dimensional case and to study the two-dimensional case [3,4]. Onsager was the first to present the exact solution of the two-dimensional Ising model on the square lattice without a magnetic field, using the transfer matrix method [5]. This famous result has since been obtained through various approaches [6,7,8,9,10,11,12,13,14], and has made a great impact on exactly solvable lattice models [15,16,17,18,19]. Lee and Yang proposed the solution for the case in an imaginary field i(ππ22)kBT in their work on Lee–Yang zeros [20]. In Ref. [20], the well-known circle theorem for Lee–Yang zeros in the complex $z=e^{-2\beta H_{\mathrm{ex}}}$ plane of the ferromagnetic Ising model was proposed, where $H_{\mathrm{ex}}$ is the field. Lee and Yang found that in addition to the zero-field case z=1, the square lattice Ising model can also be exactly solved when z=−1 [Hex=i(ππ22)kBT]. The zero-field and imaginary field cases correspond to the two points of intersection of the unit circle and the real axis in the complex *z* plane. The imaginary field solution has also been rederived using a variety of different methods [21,22,23,24,25,26,27]. Since then, mathematically exact results for Ising models have attracted considerable attention, demonstrating their elegance and significance. Two-dimensional Ising models on the honeycomb lattice [28,29,30,31], the triangular lattice [32,33,34,35], the Kagomé lattice [36,37], and the checkerboard lattice [38,39] have also been solved exactly. Although the Ising model in a non-zero field is generally a well-known unsolved problem, the case of an imaginary field i(ππ22)kBT, introduced by Lee and Yang [20], is unique and holds significant interest.

In this work, we focus on the exact solutions of anisotropic Ising models on typical two-dimensional lattices, both in the zero field and in the imaginary field. The Hamiltonian of *N* spins $\left\{s_{i}=\pm 1,i=1,\cdots ,N\right\}$ reads

(1) $$H=\sum_{\langle ij\rangle }J_{ij}s_{i}s_{j}-H_{\mathrm{ex}}\sum_{i=1}^{N}s_{i}\;,$$

where the first sum $\sum_{\langle ij\rangle }$ is over all nearest neighbors, $J_{ij}$ denotes the directional anisotropic interaction and the field $H_{\mathrm{ex}}$ can be 0 or i(ππ22)kBT. The approach we employ is the mapping into the free-fermion model [40,41,42,43,44], which was proposed in the vertex model problem.

**Figure 1:**
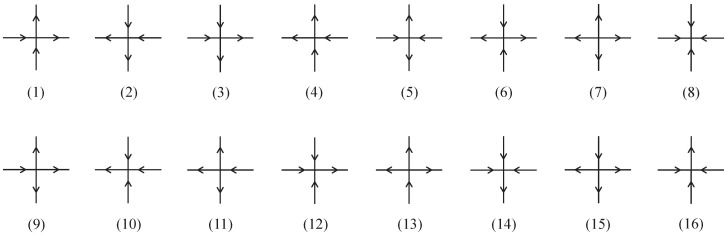
The vertex configurations of the sixteen-vertex model. The even subcase consists of vertices (**1**)–(**8**), while the odd subcase consists of vertices (**9**)–(**16**).

The vertex model is also an important and intriguing topic in statistical mechanics. Studies of vertex models such as the six-vertex [45,46,47,48,49,50,51,52,53,54,55,56], eight-vertex [40,41,42,57,58,59,60,61,62,63,64,65], and sixteen-vertex models [66,67,68,69,70,71] are closely related to those of Ising models. The free-fermion models, which are solvable eight-vertex models that satisfy the free-fermion condition, can be exactly solved using various methods such as the *S*-matrix [40], the Pfaffian [41], and the transfer matrix methods [72,73,74,75,76,77]. Typically, an eight-vertex model on the square lattice is defined as either the even or odd subcase of the general sixteen-vertex model [71], as shown in Figure 1. In this paper, we adopt the notation of vertex configurations in Ref. [68] (see Figure 1 there), and refer to the eight-vertex model of the even (odd) subcase satisfying the free-fermion condition as an even (odd) free-fermion model.

There have been lots of studies connecting the Ising model with the vertex model [26,27,78,79,80,81]. Ref. [26] introduced a rather simple and direct correspondence between the square lattice Ising spin states and the arrow configurations of the sixteen-vertex model. The value ±1 of an Ising spin is naturally mapped to the direction of the arrow settled on the corresponding site in the sixteen-vertex model. By further performing a transformation from the sixteen vertex weights to those in the even subcase, the author showed that the Ising model is equivalent with an even free-fermion model, both for the zero-field case and the imaginary field case. The present paper is inspired by the success of this work [26]. The method of transforming the square lattice Ising model to the free-fermion model is extended and applied to the Ising models on the honeycomb, triangular, and Kagomé lattices.

The structure of this paper is as follows. In Section 2, we briefly introduce the methods and techniques used for transforming the Ising models into the free-fermion models, specifically the decorated lattice technique [36,82,83], the star-triangle transformation [36,82,83,84,85], and the weak-graph expansion [68,86,87,88]. In Section 3, we present the exact results of the Ising models on the honeycomb, triangular, and Kagomé lattices. Each of these models is shown to be equivalent with a free-fermion model, both in cases under a zero field and an imaginary field. The exact solutions are then obtained in the free-fermion formulation. We summarize the study in Section 4.

## 2. Methods

It is essential to introduce the methods and techniques for the transformation of lattice systems and vertex weights before we present the main results of the Ising models. The decorated lattice technique [36,82,83] and the star-triangle transformation [16,36,82,83,84,85] help us to obtain a lattice structure, which is convenient for translating the Boltzmann factors to the vertex weights of a sixteen-vertex model. The resulting sixteen-vertex model is then transformed into an even or odd eight-vertex model by the weak-graph expansion [68,86,87,88].

### 2.1. Decorated Lattice Technique

**Figure 2:**
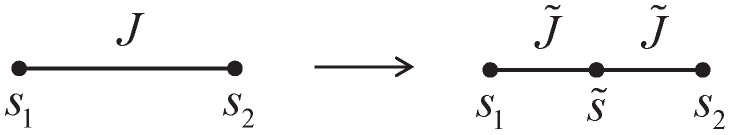
The diagrammatic representation of the decorated lattice technique. A spin $\tilde{s}$ is added and the interaction *J* is replaced with $\tilde{J}$.

The decorated lattice technique is suitable for the case when we would like to add a spin site between a pair of nearest neighbors, keeping the partition function invariant. As shown in Figure 2, interaction *J* is replaced with $\tilde{J}$. In our consideration, the additional spin $\tilde{s}$ is used to represent the direction of the arrow on the edge linking $s_{1}$ and $s_{2}$. This may help us to build up an effective vertex model formulation of the Ising problem. Consider that the Boltzmann factors of this pair contributed to the partition function, which should be conserved after the decorated lattice transformation. That is,

(2) $$e^{-\beta Js_{1}s_{2}}=A\sum_{\tilde{s}=\pm 1}e^{-\beta \tilde{J}\tilde{s}\left(s_{1}+s_{2}\right)}\;.$$

We can see clearly from Equation (2) that

$$\begin{array}{c} e^{-\beta J}=A\left(e^{-2\beta \tilde{J}}+e^{2\beta \tilde{J}}\right),\;e^{\beta J}=2A. \end{array}$$

Thus, the interaction $\tilde{J}$ and the multiplier *A* are determined by

(3) $$\cosh\left(2\beta \tilde{J}\right)=e^{-2\beta J}\;,$$

(4) $$A=\frac{1}{2}e^{\beta J}\;.$$

### 2.2. Star-Triangle Transformation

**Figure 3:**
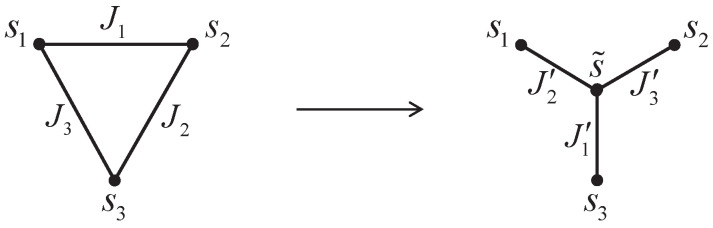
The diagrammatic representation of the star-triangle transformation. A spin $\tilde{s}$ is added, and the interactions $J_{1}$, $J_{2}$, and $J_{3}$ are replaced with $J_{1}^{\prime }$, $J_{2}^{\prime }$, and $J_{3}^{\prime }$.

Figure 3 shows the star-triangle transformation. The triangle with internal interactions $J_{1}$, $J_{2}$, and $J_{3}$ is replaced with a “star” consisting of an additional spin site coupled to the three vertices with interactions $J_{1}^{\prime }$, $J_{2}^{\prime }$, and $J_{3}^{\prime }$. The partition function should be invariant after the transformation, i.e., the Boltzmann factors of this unit should be conserved.

(5) $$e^{-\beta \left(J_{1}s_{1}s_{2}+J_{2}s_{2}s_{3}+J_{3}s_{3}s_{1}\right)}=B\sum_{\tilde{s}=\pm 1}e^{-\beta \tilde{s}\left(J_{1}^{\prime }s_{3}+J_{2}^{\prime }s_{1}+J_{3}^{\prime }s_{2}\right)}\;.$$

This leads to

$$\begin{array}{cc} \; & e^{-\beta \left(J_{1}+J_{2}+J_{3}\right)}=B\left[e^{-\beta \left(J_{1}^{\prime }+J_{2}^{\prime }+J_{3}^{\prime }\right)}+e^{-\beta \left(-J_{1}^{\prime }-J_{2}^{\prime }-J_{3}^{\prime }\right)}\right],\\ \; & e^{-\beta \left(J_{1}-J_{2}-J_{3}\right)}=B\left[e^{-\beta \left(-J_{1}^{\prime }+J_{2}^{\prime }+J_{3}^{\prime }\right)}+e^{-\beta \left(J_{1}^{\prime }-J_{2}^{\prime }-J_{3}^{\prime }\right)}\right],\\ \; & e^{-\beta \left(-J_{1}+J_{2}-J_{3}\right)}=B\left[e^{-\beta \left(J_{1}^{\prime }-J_{2}^{\prime }+J_{3}^{\prime }\right)}+e^{-\beta \left(-J_{1}^{\prime }+J_{2}^{\prime }-J_{3}^{\prime }\right)}\right],\\ \; & e^{-\beta \left(-J_{1}-J_{2}+J_{3}\right)}=B\left[e^{-\beta \left(J_{1}^{\prime }+J_{2}^{\prime }-J_{3}^{\prime }\right)}+e^{-\beta \left(-J_{1}^{\prime }-J_{2}^{\prime }+J_{3}^{\prime }\right)}\right]. \end{array}$$

The interactions $J_{1}^{\prime }$, $J_{2}^{\prime }$, and $J_{3}^{\prime }$ and multiplier *B* can be determined from the equations above [16]:

(6) $$\begin{array}{cc} \; & \cosh\left(2\beta J_{1}^{\prime }\right)=\cosh\left(2\beta J_{2}\right)\cosh\left(2\beta J_{3}\right)-\sinh\left(2\beta J_{2}\right)\sinh\left(2\beta J_{3}\right)\coth\left(2\beta J_{1}\right),\\ \; & \cosh\left(2\beta J_{2}^{\prime }\right)=\cosh\left(2\beta J_{3}\right)\cosh\left(2\beta J_{1}\right)-\sinh\left(2\beta J_{3}\right)\sinh\left(2\beta J_{1}\right)\coth\left(2\beta J_{2}\right),\\ \; & \cosh\left(2\beta J_{3}^{\prime }\right)=\cosh\left(2\beta J_{1}\right)\cosh\left(2\beta J_{2}\right)-\sinh\left(2\beta J_{1}\right)\sinh\left(2\beta J_{2}\right)\coth\left(2\beta J_{3}\right), \end{array}$$

(7) $$\begin{array}{cc} B= & \frac{\left(1-t_{1}^{2}\right)\left(1-t_{2}^{2}\right)\left(1-t_{3}^{2}\right)}{4}{\left[\frac{-\sinh\left(2\beta J_{1}\right)\sinh\left(2\beta J_{2}\right)\sinh\left(2\beta J_{3}\right)}{2\left(1-t_{1}t_{2}t_{3}\right)\left(t_{2}t_{3}-t_{1}\right)\left(t_{1}t_{3}-t_{2}\right)\left(t_{1}t_{2}-t_{3}\right)}\right]}^{1/2}, \end{array}$$

with $t_{i}=\tanh\left(\beta J_{i}\right)\left(i=1,2,3\right)$. One can easily verify that the star-triangle transformation directly connects the partition function of the triangular lattice model with that of the honeycomb lattice model. This enables us to simplify the derivation of the results for the triangular lattice model.

### 2.3. Weak-Graph Expansion

Weak-graph expansion is a useful tool in lattice statistical problems [86,88]. In this paper, we use the version of the weak-graph expansion method proposed by Wu, which was originally designed for the even eight-vertex model [87] and later extended to the sixteen-vertex model [68]. Applying the method to the sixteen-vertex model enables us to obtain a new set of vertex weights, with the partition function invariant. The details of the method are shown in Ref. [87], and we only list the result for all sixteen rearranged weights, expressed via a transformation matrix M as ${\left\{{\tilde{\omega }}_{1},{\tilde{\omega }}_{2},\ldots ,{\tilde{\omega }}_{16}\right\}}^{T}=\frac{1}{4}M{\left\{\omega_{1},\omega_{2},\ldots ,\omega_{16}\right\}}^{T}$, with

(8) $$\begin{array}{c} M=\left(\begin{array}{cccccccccccccccc} 1 & 1 & 1 & 1 & 1 & 1 & 1 & 1 & 1 & 1 & 1 & 1 & 1 & 1 & 1 & 1\\ 1 & 1 & 1 & 1 & 1 & 1 & 1 & 1 & -1 & -1 & -1 & -1 & -1 & -1 & -1 & -1\\ 1 & 1 & 1 & 1 & -1 & -1 & -1 & -1 & -1 & -1 & -1 & -1 & 1 & 1 & 1 & 1\\ 1 & 1 & 1 & 1 & -1 & -1 & -1 & -1 & 1 & 1 & 1 & 1 & -1 & -1 & -1 & -1\\ 1 & 1 & -1 & -1 & 1 & 1 & -1 & -1 & -1 & -1 & 1 & 1 & 1 & 1 & -1 & -1\\ 1 & 1 & -1 & -1 & 1 & 1 & -1 & -1 & 1 & 1 & -1 & -1 & -1 & -1 & 1 & 1\\ 1 & 1 & -1 & -1 & -1 & -1 & 1 & 1 & -1 & -1 & 1 & 1 & -1 & -1 & 1 & 1\\ 1 & 1 & -1 & -1 & -1 & -1 & 1 & 1 & 1 & 1 & -1 & -1 & 1 & 1 & -1 & -1\\ -1 & 1 & 1 & -1 & 1 & -1 & 1 & -1 & 1 & -1 & 1 & -1 & -1 & 1 & 1 & -1\\ -1 & 1 & 1 & -1 & 1 & -1 & 1 & -1 & -1 & 1 & -1 & 1 & 1 & -1 & -1 & 1\\ -1 & 1 & 1 & -1 & -1 & 1 & -1 & 1 & 1 & -1 & 1 & -1 & 1 & -1 & -1 & 1\\ -1 & 1 & 1 & -1 & -1 & 1 & -1 & 1 & -1 & 1 & -1 & 1 & -1 & 1 & 1 & -1\\ -1 & 1 & -1 & 1 & -1 & 1 & 1 & -1 & -1 & 1 & 1 & -1 & 1 & -1 & 1 & -1\\ -1 & 1 & -1 & 1 & -1 & 1 & 1 & -1 & 1 & -1 & -1 & 1 & -1 & 1 & -1 & 1\\ -1 & 1 & -1 & 1 & 1 & -1 & -1 & 1 & 1 & -1 & -1 & 1 & 1 & -1 & 1 & -1\\ -1 & 1 & -1 & 1 & 1 & -1 & -1 & 1 & -1 & 1 & 1 & -1 & -1 & 1 & -1 & 1 \end{array}\right). \end{array}$$

The sixteen-vertex model is symmetric when the weight of each vertex configuration is invariant under the reversal of arrows, i.e.,

(9) $$\omega_{1}=\omega_{2},\ldots ,\omega_{15}=\omega_{16}.$$

Similarly, the antisymmetric sixteen-vertex model satisfies

(10) $$\omega_{1}=-\omega_{2},\ldots ,\omega_{15}=-\omega_{16}.$$

It is straightforward to see from Equation (8) that the symmetric sixteen-vertex model is transformed into an even eight-vertex model (Equation (3) of Ref. [68] shows this case), while the antisymmetric case is transformed into an odd subcase.

We will show in the next section that each Ising model considered can be mapped to a symmetric/antisymmetric sixteen-vertex model, and further transformed into an even/odd eight-vertex model by Equation (8). We can find that each case satisfies either the even free-fermion condition [40,41]

(11) $${\tilde{\omega }}_{1}{\tilde{\omega }}_{2}+{\tilde{\omega }}_{3}{\tilde{\omega }}_{4}={\tilde{\omega }}_{5}{\tilde{\omega }}_{6}+{\tilde{\omega }}_{7}{\tilde{\omega }}_{8}$$

or the odd free-fermion condition [42]

(12) $${\tilde{\omega }}_{9}{\tilde{\omega }}_{10}+{\tilde{\omega }}_{11}{\tilde{\omega }}_{12}={\tilde{\omega }}_{13}{\tilde{\omega }}_{14}+{\tilde{\omega }}_{15}{\tilde{\omega }}_{16}\;.$$

## 3. Results

### 3.1. The Honeycomb Lattice

**Figure 4:**
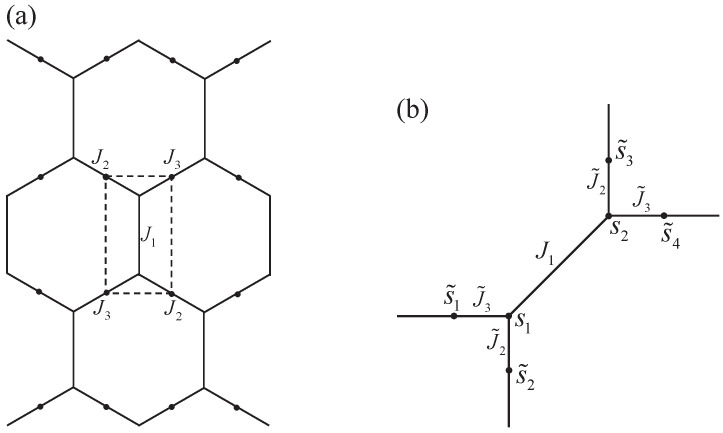
The diagrammatic representation of applying the decorated lattice technique to the honeycomb lattice model. The region bounded by dashed lines in (**a**) forms a vertex site of the sixteen-vertex model. The details of the vertex unit are shown in (**b**).

We apply the decorated lattice technique to the honeycomb lattice, as shown in Figure 4a. It is easy to verify that the region bounded by dash lines can be regarded as a vertex site on the square lattice. Each vertex site consists of two honeycomb lattice Ising spins. Figure 4b shows the details with ${\tilde{J}}_{2}$ and ${\tilde{J}}_{3}$ determined from $J_{2}$ and $J_{3}$ by Equation (3), respectively. It is natural to introduce a one-to-one mapping from the spin states of $\left\{\tilde{s}\right\}$ in Figure 4b to the arrow configurations

(13) $$\begin{array}{ccc} & & \mathrm{for}\;\mathrm{horizontal}\;\mathrm{edge}\;\;\frac{\;\;}{\;\;}\;\;•\;\;\frac{\;\;}{\;\;}\;+/-\;\Rightarrow \;\to /\leftarrow ,\\ & & \mathrm{for}\;\mathrm{vertical}\;\mathrm{edge}\;\;\overset{|}{\underset{|}{•}}\;+/-\;\Rightarrow \;↑/↓. \end{array}$$

Therefore, a sixteen-vertex model can be built up with an appropriate correspondence between the vertex weights and the Boltzmann factors of the Ising model.

#### 3.1.1. Zero-Field Case

In the zero-field case, the partition function can be expressed by

(14) $$Z=A_{2}^{N/2}A_{3}^{N/2}\sum_{\left\{\tilde{s}\right\}=\pm 1}\;\prod_{\mathrm{all}\;\mathrm{vertices}}\omega \left({\tilde{s}}_{1},{\tilde{s}}_{2},{\tilde{s}}_{3},{\tilde{s}}_{4}\right)$$

with

(15) $$\begin{array}{cc} \; & \omega \left({\tilde{s}}_{1},{\tilde{s}}_{2},{\tilde{s}}_{3},{\tilde{s}}_{4}\right)=\sum_{s_{1}=\pm 1,s_{2}=\pm 1}e^{-\beta J_{1}s_{1}s_{2}}e^{-\beta \left[{\tilde{J}}_{2}\left(s_{1}{\tilde{s}}_{2}+s_{2}{\tilde{s}}_{3}\right)+{\tilde{J}}_{3}\left(s_{1}{\tilde{s}}_{1}+s_{2}{\tilde{s}}_{4}\right)\right]} \end{array}$$

and $A_{i}$ determined from $J_{i}$ by Equation (4). $\omega \left({\tilde{s}}_{1},{\tilde{s}}_{2},{\tilde{s}}_{3},{\tilde{s}}_{4}\right)$ is the Boltzmann factor of the vertex unit shown in Figure 4b. Then, the vertex weights can be determined directly from $\omega \left({\tilde{s}}_{1},{\tilde{s}}_{2},{\tilde{s}}_{3},{\tilde{s}}_{4}\right)$, according to the vertex configurations in Figure 1 and the mapping in Equation (13). That is,

(16) $$\begin{array}{cc} \; & \omega_{1}=\omega \left(++++\right)=2e^{\beta J_{1}}+e^{-\beta J_{1}}\left[e^{2\beta \left({\tilde{J}}_{2}+{\tilde{J}}_{3}\right)}+e^{-2\beta \left({\tilde{J}}_{2}+{\tilde{J}}_{3}\right)}\right],\\ \; & \omega_{2}=\omega_{1},\\ \; & \omega_{3}=\omega \left(+--+\right)=2e^{\beta J_{1}}+e^{-\beta J_{1}}\left[e^{2\beta \left({\tilde{J}}_{2}-{\tilde{J}}_{3}\right)}+e^{-2\beta \left({\tilde{J}}_{2}-{\tilde{J}}_{3}\right)}\right],\\ \; & \omega_{4}=\omega_{3},\\ \; & \omega_{5}=\omega \left(+-+-\right)=2e^{-\beta J_{1}}+e^{\beta J_{1}}\left[e^{2\beta \left({\tilde{J}}_{2}-{\tilde{J}}_{3}\right)}+e^{-2\beta \left({\tilde{J}}_{2}-{\tilde{J}}_{3}\right)}\right],\\ \; & \omega_{6}=\omega_{5},\\ \; & \omega_{7}=\omega \left(--++\right)=2e^{-\beta J_{1}}+e^{\beta J_{1}}\left[e^{2\beta \left({\tilde{J}}_{2}+{\tilde{J}}_{3}\right)}+e^{-2\beta \left({\tilde{J}}_{2}+{\tilde{J}}_{3}\right)}\right],\\ \; & \omega_{8}=\omega_{7},\\ \; & \omega_{9}=\omega \left(+-++\right)=e^{\beta J_{1}}\left(e^{2\beta {\tilde{J}}_{2}}+e^{-2\beta {\tilde{J}}_{2}}\right)+e^{-\beta J_{1}}\left(e^{2\beta {\tilde{J}}_{3}}+e^{-2\beta {\tilde{J}}_{3}}\right),\\ \; & \omega_{10}=\omega_{11}=\omega_{12}=\omega_{9},\\ \; & \omega_{13}=\omega \left(-+++\right)=e^{-\beta J_{1}}\left(e^{2\beta {\tilde{J}}_{2}}+e^{-2\beta {\tilde{J}}_{2}}\right)+e^{\beta J_{1}}\left(e^{2\beta {\tilde{J}}_{3}}+e^{-2\beta {\tilde{J}}_{3}}\right),\\ \; & \omega_{14}=\omega_{15}=\omega_{16}=\omega_{13}. \end{array}$$

We see that the resulting sixteen-vertex model is symmetric. As stated before, the vertex weights can be rearranged by performing the weak-graph expansion such that an even eight-vertex model is obtained. The new weights are straightforward to show from Equation (8):

(17) $$\begin{array}{cc} \; & {\tilde{\omega }}_{1}=\frac{1}{2}\omega_{1}+\frac{1}{2}\omega_{3}+\frac{1}{2}\omega_{5}+\frac{1}{2}\omega_{7}+\omega_{9}+\omega_{13}=2\left(e^{\beta J_{1}}+e^{-\beta J_{1}}\right)\left(e^{-2\beta J_{2}}+1\right)\left(e^{-2\beta J_{3}}+1\right),\\ \; & {\tilde{\omega }}_{2}=\frac{1}{2}\omega_{1}+\frac{1}{2}\omega_{3}+\frac{1}{2}\omega_{5}+\frac{1}{2}\omega_{7}-\omega_{9}-\omega_{13}=2\left(e^{\beta J_{1}}+e^{-\beta J_{1}}\right)\left(e^{-2\beta J_{2}}-1\right)\left(e^{-2\beta J_{3}}-1\right),\\ \; & {\tilde{\omega }}_{3}=\frac{1}{2}\omega_{1}+\frac{1}{2}\omega_{3}-\frac{1}{2}\omega_{5}-\frac{1}{2}\omega_{7}-\omega_{9}+\omega_{13}=2\left(e^{\beta J_{1}}-e^{-\beta J_{1}}\right)\left(-e^{-2\beta J_{2}}+1\right)\left(e^{-2\beta J_{3}}+1\right),\\ \; & {\tilde{\omega }}_{4}=\frac{1}{2}\omega_{1}+\frac{1}{2}\omega_{3}-\frac{1}{2}\omega_{5}-\frac{1}{2}\omega_{7}+\omega_{9}-\omega_{13}=2\left(e^{\beta J_{1}}-e^{-\beta J_{1}}\right)\left(e^{-2\beta J_{2}}+1\right)\left(-e^{-2\beta J_{3}}+1\right),\\ \; & {\tilde{\omega }}_{5}=\frac{1}{2}\omega_{1}-\frac{1}{2}\omega_{3}+\frac{1}{2}\omega_{5}-\frac{1}{2}\omega_{7}=-2\left(e^{\beta J_{1}}-e^{-\beta J_{1}}\right){\left(e^{-4\beta J_{2}}-1\right)}^{1/2}{\left(e^{-4\beta J_{3}}-1\right)}^{1/2},\\ \; & {\tilde{\omega }}_{6}={\tilde{\omega }}_{5},\\ \; & {\tilde{\omega }}_{7}=\frac{1}{2}\omega_{1}-\frac{1}{2}\omega_{3}-\frac{1}{2}\omega_{5}+\frac{1}{2}\omega_{7}=2\left(e^{\beta J_{1}}+e^{-\beta J_{1}}\right){\left(e^{-4\beta J_{2}}-1\right)}^{1/2}{\left(e^{-4\beta J_{3}}-1\right)}^{1/2},\\ \; & {\tilde{\omega }}_{8}={\tilde{\omega }}_{7}. \end{array}$$

One can easily examine that the even the free-fermion condition of Equation (11) is satisfied. Thus, the honeycomb lattice Ising model in the zero field is equivalent with an even free-fermion model. Denote the number of vertex sites by $N_{v}$. The exact solution of the even free-fermion model $\lim_{N_{v}\to \infty }\frac{1}{N_{v}}\ln Z_{\mathrm{even}}$ is given in Refs. [40,41] (note that the ordering of vertices (5)–(8) in Figure 1 differs slightly from that of Refs. [40,41], but the expression for the solution is the same). We show the result, quoting this well-known solution and noticing Equation (14)

(18) $$\begin{array}{cc} \; & \;\;\;\;\;\lim_{N\to \infty }\frac{1}{N}\ln Z\\ \; & =\frac{1}{2}\left(\ln A_{2}+\ln A_{3}\right)+\frac{1}{2}\lim_{N_{v}\to \infty }\frac{1}{N_{v}}\ln Z_{\mathrm{even}}\\ \; & =\frac{1}{2}\ln\left(\frac{1}{2}e^{\beta J_{2}}\right)+\frac{1}{2}\ln\left(\frac{1}{2}e^{\beta J_{3}}\right)+\frac{1}{2}\frac{1}{8\pi^{2}}\int_{0}^{2\pi }d\theta \int_{0}^{2\pi }d\phi \ln\left[a+b\cos\theta +c\cos\phi +d\cos\left(\theta -\phi \right)+e\cos\left(\theta +\phi \right)\right] \end{array}$$

with

(19) $$\begin{array}{cc} \; & a={\tilde{\omega }}_{1}^{2}+{\tilde{\omega }}_{2}^{2}+{\tilde{\omega }}_{3}^{2}+{\tilde{\omega }}_{4}^{2},\\ \; & b=2({\tilde{\omega }}_{1}{\tilde{\omega }}_{3}-{\tilde{\omega }}_{2}{\tilde{\omega }}_{4}),\\ \; & c=2({\tilde{\omega }}_{1}{\tilde{\omega }}_{4}-{\tilde{\omega }}_{2}{\tilde{\omega }}_{3}),\\ \; & d=2({\tilde{\omega }}_{3}{\tilde{\omega }}_{4}-{\tilde{\omega }}_{7}{\tilde{\omega }}_{8}),\\ \; & e=2({\tilde{\omega }}_{3}{\tilde{\omega }}_{4}-{\tilde{\omega }}_{5}{\tilde{\omega }}_{6}). \end{array}$$

A straightforward calculation of the coefficients in Equation (19) from the vertex weights in Equation (17) gives

(20) $$\begin{array}{cc} \; & a=2^{7}e^{-2\beta (J_{2}+J_{3})}\left[\cosh\left(2\beta J_{1}\right)\cosh\left(2\beta J_{2}\right)\cosh\left(2\beta J_{3}\right)+1\right],\\ \; & b=2^{7}e^{-2\beta (J_{2}+J_{3})}\sinh\left(2\beta J_{1}\right)\sinh\left(2\beta J_{2}\right),\\ \; & c=2^{7}e^{-2\beta (J_{2}+J_{3})}\sinh\left(2\beta J_{1}\right)\sinh\left(2\beta J_{3}\right),\\ \; & d=-2^{7}e^{-2\beta (J_{2}+J_{3})}\sinh\left(2\beta J_{2}\right)\sinh\left(2\beta J_{3}\right),\\ \; & e=0. \end{array}$$

After inserting Equation (20) into Equation (18) and making some arrangements, the exact solution turns out to be

(21) $$\begin{array}{cc} \; & \lim_{N\to \infty }\frac{1}{N}\ln Z=\frac{3}{4}\ln2+\frac{1}{16\pi^{2}}\int_{0}^{2\pi }d\theta \int_{0}^{2\pi }d\phi \ln\left[\cosh\left(2\beta J_{1}\right)\cosh\left(2\beta J_{2}\right)\cosh\left(2\beta J_{3}\right)+1\right.\\ \; & \;\;\;\;\;\;\;\;\;\;\;\;\;\;\;\;\;\;\;\;\;\;\;\;\;\;\;\;\;\;\;\;\;\;\;\;\;\;\;+\sinh\left(2\beta J_{1}\right)\sinh\left(2\beta J_{2}\right)\cos\theta +\sinh\left(2\beta J_{1}\right)\sinh\left(2\beta J_{3}\right)\cos\phi \\ \; & \;\;\;\;\;\;\;\;\;\;\;\;\;\;\;\;\;\;\;\;\;\;\;\;\;\;\;\;\;\;\;\;\;\;\;\;\;\;\;\left.-\sinh\left(2\beta J_{2}\right)\sinh\left(2\beta J_{3}\right)\cos\left(\theta -\phi \right)\right]. \end{array}$$

One can verify that this expression is consistent with the results published in previous studies [28,29,30,31].

#### 3.1.2. Imaginary Field Case

In the case of Hex=i(ππ22)kBT, we notice that

(22) $$e^{\beta H_{\mathrm{ex}}\sum_{i=1}^{N}s_{i}}=i^{\sum_{i=1}^{N}s_{i}}=i^{N}\prod_{i=1}^{N}s_{i}$$

using the identity $i^{s_{i}}=i\times s_{i}$ [27]. We can see that $i^{N}$ is a constant, and the product $\prod_{i=1}^{N}s_{i}$ can be conveniently included in the factors $\omega \left({\tilde{s}}_{1},{\tilde{s}}_{2},{\tilde{s}}_{3},{\tilde{s}}_{4}\right)$. We just need to rewrite the partition function as

(23) $$Z=i^{N}A_{2}^{N/2}A_{3}^{N/2}\sum_{\left\{\tilde{s}\right\}=\pm 1}\;\prod_{\mathrm{all}\;\mathrm{vertices}}\omega \left({\tilde{s}}_{1},{\tilde{s}}_{2},{\tilde{s}}_{3},{\tilde{s}}_{4}\right)$$

with

(24) $$\begin{array}{cc} \; & \omega \left({\tilde{s}}_{1},{\tilde{s}}_{2},{\tilde{s}}_{3},{\tilde{s}}_{4}\right)=\sum_{s_{1}=\pm 1,s_{2}=\pm 1}\left(s_{1}s_{2}\right)e^{-\beta J_{1}s_{1}s_{2}}e^{-\beta \left[{\tilde{J}}_{2}\left(s_{1}{\tilde{s}}_{2}+s_{2}{\tilde{s}}_{3}\right)+{\tilde{J}}_{3}\left(s_{1}{\tilde{s}}_{1}+s_{2}{\tilde{s}}_{4}\right)\right]}. \end{array}$$

The vertex weights are then determined in the similar way to those in Equation (16):

(25) $$\begin{array}{cc} \; & \omega_{1}=\omega_{2}=-2e^{\beta J_{1}}+e^{-\beta J_{1}}\left[e^{2\beta \left({\tilde{J}}_{2}+{\tilde{J}}_{3}\right)}+e^{-2\beta \left({\tilde{J}}_{2}+{\tilde{J}}_{3}\right)}\right],\\ \; & \omega_{3}=\omega_{4}=-2e^{\beta J_{1}}+e^{-\beta J_{1}}\left[e^{2\beta \left({\tilde{J}}_{2}-{\tilde{J}}_{3}\right)}+e^{-2\beta \left({\tilde{J}}_{2}-{\tilde{J}}_{3}\right)}\right],\\ \; & \omega_{5}=\omega_{6}=2e^{-\beta J_{1}}-e^{\beta J_{1}}\left[e^{2\beta \left({\tilde{J}}_{2}-{\tilde{J}}_{3}\right)}+e^{-2\beta \left({\tilde{J}}_{2}-{\tilde{J}}_{3}\right)}\right],\\ \; & \omega_{7}=\omega_{8}=2e^{-\beta J_{1}}-e^{\beta J_{1}}\left[e^{2\beta \left({\tilde{J}}_{2}+{\tilde{J}}_{3}\right)}+e^{-2\beta \left({\tilde{J}}_{2}+{\tilde{J}}_{3}\right)}\right],\\ \; & \omega_{9}=\omega_{10}=\omega_{11}=\omega_{12}=-e^{\beta J_{1}}\left(e^{2\beta {\tilde{J}}_{2}}+e^{-2\beta {\tilde{J}}_{2}}\right)+e^{-\beta J_{1}}\left(e^{2\beta {\tilde{J}}_{3}}+e^{-2\beta {\tilde{J}}_{3}}\right),\\ \; & \omega_{13}=\omega_{14}=\omega_{15}=\omega_{16}=e^{-\beta J_{1}}\left(e^{2\beta {\tilde{J}}_{2}}+e^{-2\beta {\tilde{J}}_{2}}\right)-e^{\beta J_{1}}\left(e^{2\beta {\tilde{J}}_{3}}+e^{-2\beta {\tilde{J}}_{3}}\right). \end{array}$$

Again, the Boltzmann factors are translated into the weights of a symmetric sixteen-vertex model. We still perform the weak-graph expansion via Equation (8):

(26) $$\begin{array}{cc} {\tilde{\omega }}_{1} & =2\left(-e^{\beta J_{1}}+e^{-\beta J_{1}}\right)\left(e^{-2\beta J_{2}}+1\right)\left(e^{-2\beta J_{3}}+1\right),\\ {\tilde{\omega }}_{2} & =2\left(-e^{\beta J_{1}}+e^{-\beta J_{1}}\right)\left(e^{-2\beta J_{2}}-1\right)\left(e^{-2\beta J_{3}}-1\right),\\ {\tilde{\omega }}_{3} & =-2\left(e^{\beta J_{1}}+e^{-\beta J_{1}}\right)\left(-e^{-2\beta J_{2}}+1\right)\left(e^{-2\beta J_{3}}+1\right),\\ {\tilde{\omega }}_{4} & =-2\left(e^{\beta J_{1}}+e^{-\beta J_{1}}\right)\left(e^{-2\beta J_{2}}+1\right)\left(-e^{-2\beta J_{3}}+1\right),\\ {\tilde{\omega }}_{5} & ={\tilde{\omega }}_{6}=2\left(e^{\beta J_{1}}+e^{-\beta J_{1}}\right){\left(e^{-4\beta J_{2}}-1\right)}^{1/2}{\left(e^{-4\beta J_{3}}-1\right)}^{1/2},\\ {\tilde{\omega }}_{7} & ={\tilde{\omega }}_{8}=2\left(-e^{\beta J_{1}}+e^{-\beta J_{1}}\right){\left(e^{-4\beta J_{2}}-1\right)}^{1/2}{\left(e^{-4\beta J_{3}}-1\right)}^{1/2}. \end{array}$$

Clearly, we obtained an even free-fermion model again. Notice Equation (23), and the partition function is

(27) $$\lim_{N\to \infty }\frac{1}{N}\ln Z=i\frac{\pi }{2}+\frac{1}{2}\left(\ln A_{2}+\ln A_{3}\right)+\frac{1}{2}\lim_{N_{v}\to \infty }\frac{1}{N_{v}}\ln Z_{\mathrm{even}}$$

with the even free-fermion solution expressed in Equations (18) and (19). Omitting the detailed calculations, we show the final exact result:

(28) $$\begin{array}{cc} \; & \lim_{N\to \infty }\frac{1}{N}\ln Z=i\frac{\pi }{2}+\frac{3}{4}\ln2+\frac{1}{16\pi^{2}}\int_{0}^{2\pi }d\theta \int_{0}^{2\pi }d\phi \ln\left[\cosh\left(2\beta J_{1}\right)\cosh\left(2\beta J_{2}\right)\cosh\left(2\beta J_{3}\right)-1\right.\\ \; & \;\;\;\;\;\;\;\;\;\;\;\;\;\;\;\;\;\;\;\;\;\;\;\;\;\;\;\;\;\;\;\;\;\;\;\;\;\;\;\;\;+\sinh\left(2\beta J_{1}\right)\sinh\left(2\beta J_{2}\right)\cos\theta +\sinh\left(2\beta J_{1}\right)\sinh\left(2\beta J_{3}\right)\cos\phi \\ \; & \;\;\;\;\;\;\;\;\;\;\;\;\;\;\;\;\;\;\;\;\;\;\;\;\;\;\;\;\;\;\;\;\;\;\;\;\;\;\;\;\;\left.+\sinh\left(2\beta J_{2}\right)\sinh\left(2\beta J_{3}\right)\cos\left(\theta -\phi \right)\right]. \end{array}$$

One can examine that our result recovers the proposed solutions of this case [21,27].

### 3.2. The Triangular Lattice

**Figure 5:**
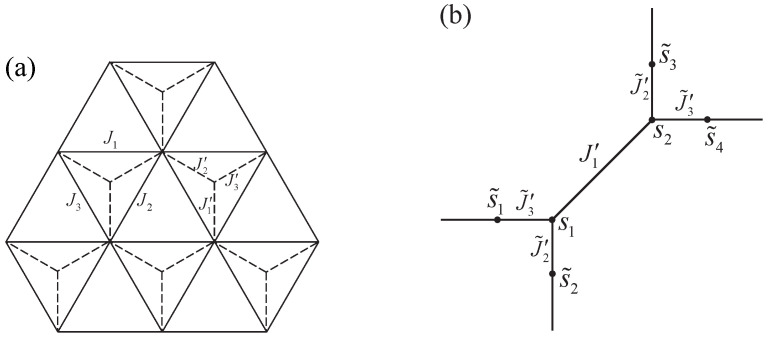
The diagrammatic representation of applying the star-triangle transformation to the triangular lattice model. The transformed system, which is on the honeycomb lattice as shown by the dash lines in (**a**), can be mapped into the sixteen-vertex model in the same way as that in Figure 4a. The details of the vertex unit are shown in (**b**).

To deal with the triangular lattice Ising model, we employ the star-triangle transformation as shown in Figure 5a. The transformed spin system is on the honeycomb lattice, with the replacement of the nearest neighbor interactions $J_{i}\to J_{i}^{\prime }$. The new anisotropic interactions $J_{i}^{\prime }$ are determined by Equation (6). Applying the decorated lattice technique in the same way as that in Figure 4a, we construct a sixteen-vertex model of which the vertex site is shown Figure 5b. The mapping from the spin states to the arrow configurations is still that in Equation (13). The relation between $J_{i}^{\prime }$ and ${{\tilde{J}}^{\prime }}_{i}$ is the same with that in Equation (3).

#### 3.2.1. Zero-Field Case

In the zero field, the partition function can be obtained using the result for the honeycomb lattice as

$$\begin{array}{c} Z=B^{N}Z\left(\mathrm{honeycomb},J_{i}\to J_{i}^{\prime },H_{\mathrm{ex}}=0\right) \end{array}$$

with *B* defined in Equation (7). Obviously this case is also equivalent with an even free-fermion model. Since the number of spins on the transformed honeycomb lattice is double that on the triangular lattice, the partition function should be

(29) $$\lim_{N\to \infty }\frac{1}{N}\ln Z=\ln B+2\times \mathrm{Equation}\;\left(21\right)\;\left(J_{i}\to J_{i}^{\prime }\right).$$

Taking into account Equations (6), (7) and (21), the exact solution can be obtained

(30) $$\begin{array}{cc} \lim_{N\to \infty }\frac{1}{N}\ln Z= & \ln2+\frac{1}{8\pi^{2}}\int_{0}^{2\pi }d\theta \int_{0}^{2\pi }d\phi \ln\left[\cosh\left(2\beta J_{1}\right)\cosh\left(2\beta J_{2}\right)\cosh\left(2\beta J_{3}\right)\right.\\ & -\sinh\left(2\beta J_{1}\right)\sinh\left(2\beta J_{2}\right)\sinh\left(2\beta J_{3}\right)-\sinh\left(2\beta J_{3}\right)\cos\theta -\sinh\left(2\beta J_{2}\right)\cos\phi \\ & \left.+\sinh\left(2\beta J_{1}\right)\cos\left(\theta -\phi \right)\right]. \end{array}$$

This expression agrees with the known results of this case [32,33,34].

#### 3.2.2. Imaginary Field Case

We still make use of Equation (22) to deal with the effect of the imaginary field. The partition function of this case should be

(31) $$Z=i^{N}B^{N}{A_{2}^{\prime }}^{N}{A_{3}^{\prime }}^{N}\sum_{\left\{\tilde{s}\right\}=\pm 1}\;\prod_{\mathrm{all}\;\mathrm{vertices}}\omega \left({\tilde{s}}_{1},{\tilde{s}}_{2},{\tilde{s}}_{3},{\tilde{s}}_{4}\right)$$

with $A_{i}^{\prime }=\frac{1}{2}e^{\beta J_{i}^{\prime }}$ and

(32) $$\begin{array}{cc} \; & \omega \left({\tilde{s}}_{1},{\tilde{s}}_{2},{\tilde{s}}_{3},{\tilde{s}}_{4}\right)=\sum_{s_{1}=\pm 1,s_{2}=\pm 1}\left(s_{1}\right)e^{-\beta J_{1}^{\prime }s_{1}s_{2}}e^{-\beta \left[{{\tilde{J}}^{\prime }}_{2}\left(s_{1}{\tilde{s}}_{2}+s_{2}{\tilde{s}}_{3}\right)+{{\tilde{J}}^{\prime }}_{3}\left(s_{1}{\tilde{s}}_{1}+s_{2}{\tilde{s}}_{4}\right)\right]}. \end{array}$$

Note that factors $\omega \left({\tilde{s}}_{1},{\tilde{s}}_{2},{\tilde{s}}_{3},{\tilde{s}}_{4}\right)$ differ from those in Equation (24) as the spin $s_{2}$ is not on the original triangular lattice. Similarly, factors $\omega \left({\tilde{s}}_{1},{\tilde{s}}_{2},{\tilde{s}}_{3},{\tilde{s}}_{4}\right)$ are translated into the vertex weights of a sixteen-vertex model. We find that the vertex weights in this case are antisymmetric:

(33) $$\begin{array}{cc} \; & \omega_{1}=e^{-\beta J_{1}^{\prime }}\left[e^{-2\beta \left({{\tilde{J}}^{\prime }}_{2}+{{\tilde{J}}^{\prime }}_{3}\right)}-e^{2\beta \left({{\tilde{J}}^{\prime }}_{2}+{{\tilde{J}}^{\prime }}_{3}\right)}\right],\\ \; & \omega_{2}=-\omega_{1},\\ \; & \omega_{3}=e^{-\beta J_{1}^{\prime }}\left[e^{2\beta \left({{\tilde{J}}^{\prime }}_{2}-{{\tilde{J}}^{\prime }}_{3}\right)}-e^{-2\beta \left({{\tilde{J}}^{\prime }}_{2}-{{\tilde{J}}^{\prime }}_{3}\right)}\right],\\ \; & \omega_{4}=-\omega_{3},\\ \; & \omega_{5}=e^{\beta J_{1}^{\prime }}\left[e^{2\beta \left({{\tilde{J}}^{\prime }}_{2}-{{\tilde{J}}^{\prime }}_{3}\right)}-e^{-2\beta \left({{\tilde{J}}^{\prime }}_{2}-{{\tilde{J}}^{\prime }}_{3}\right)}\right],\\ \; & \omega_{6}=-\omega_{5},\\ \; & \omega_{7}=e^{\beta J_{1}^{\prime }}\left[e^{2\beta \left({{\tilde{J}}^{\prime }}_{2}+{{\tilde{J}}^{\prime }}_{3}\right)}-e^{-2\beta \left({{\tilde{J}}^{\prime }}_{2}+{{\tilde{J}}^{\prime }}_{3}\right)}\right],\\ \; & \omega_{8}=-\omega_{7},\\ \; & \omega_{9}=e^{\beta J_{1}^{\prime }}\left(e^{2\beta {{\tilde{J}}^{\prime }}_{2}}-e^{-2\beta {{\tilde{J}}^{\prime }}_{2}}\right)+e^{-\beta J_{1}^{\prime }}\left(e^{-2\beta {{\tilde{J}}^{\prime }}_{3}}-e^{2\beta {{\tilde{J}}^{\prime }}_{3}}\right),\\ \; & \omega_{10}=-\omega_{9},\\ \; & \omega_{11}=e^{\beta J_{1}^{\prime }}\left(e^{2\beta {{\tilde{J}}^{\prime }}_{2}}-e^{-2\beta {{\tilde{J}}^{\prime }}_{2}}\right)+e^{-\beta J_{1}^{\prime }}\left(e^{2\beta {{\tilde{J}}^{\prime }}_{3}}-e^{-2\beta {{\tilde{J}}^{\prime }}_{3}}\right),\\ \; & \omega_{12}=-\omega_{11},\\ \; & \omega_{13}=e^{-\beta J_{1}^{\prime }}\left(e^{-2\beta {{\tilde{J}}^{\prime }}_{2}}-e^{2\beta {{\tilde{J}}^{\prime }}_{2}}\right)+e^{\beta J_{1}^{\prime }}\left(e^{2\beta {{\tilde{J}}^{\prime }}_{3}}-e^{-2\beta {{\tilde{J}}^{\prime }}_{3}}\right),\\ \; & \omega_{14}=-\omega_{13},\\ \; & \omega_{15}=e^{-\beta J_{1}^{\prime }}\left(e^{2\beta {{\tilde{J}}^{\prime }}_{2}}-e^{-2\beta {{\tilde{J}}^{\prime }}_{2}}\right)+e^{\beta J_{1}^{\prime }}\left(e^{2\beta {{\tilde{J}}^{\prime }}_{3}}-e^{-2\beta {{\tilde{J}}^{\prime }}_{3}}\right),\\ \; & \omega_{16}=-\omega_{15}. \end{array}$$

From these weights, the weak-graph expansion produces an odd eight-vertex model with Equation (8):

(34) $$\begin{array}{cc} \; & {\tilde{\omega }}_{9}=2\cosh\left(\beta J_{1}^{\prime }\right)\left\{\sinh\left[2\beta \left({{\tilde{J}}^{\prime }}_{2}+{{\tilde{J}}^{\prime }}_{3}\right)\right]+\sinh\left[2\beta \left({{\tilde{J}}^{\prime }}_{2}-{{\tilde{J}}^{\prime }}_{3}\right)\right]+2\sinh\left(2\beta {{\tilde{J}}^{\prime }}_{2}\right)\right\},\\ \; & {\tilde{\omega }}_{10}=2\cosh\left(\beta J_{1}^{\prime }\right)\left\{\sinh\left[2\beta \left({{\tilde{J}}^{\prime }}_{2}+{{\tilde{J}}^{\prime }}_{3}\right)\right]+\sinh\left[2\beta \left({{\tilde{J}}^{\prime }}_{2}-{{\tilde{J}}^{\prime }}_{3}\right)\right]-2\sinh\left(2\beta {{\tilde{J}}^{\prime }}_{2}\right)\right\},\\ \; & {\tilde{\omega }}_{11}=-2\sinh\left(\beta J_{1}^{\prime }\right)\left\{\sinh\left[2\beta \left({{\tilde{J}}^{\prime }}_{2}+{{\tilde{J}}^{\prime }}_{3}\right)\right]+\sinh\left[2\beta \left({{\tilde{J}}^{\prime }}_{2}-{{\tilde{J}}^{\prime }}_{3}\right)\right]-2\sinh\left(2\beta {{\tilde{J}}^{\prime }}_{2}\right)\right\},\\ \; & {\tilde{\omega }}_{12}=-2\sinh\left(\beta J_{1}^{\prime }\right)\left\{\sinh\left[2\beta \left({{\tilde{J}}^{\prime }}_{2}+{{\tilde{J}}^{\prime }}_{3}\right)\right]+\sinh\left[2\beta \left({{\tilde{J}}^{\prime }}_{2}-{{\tilde{J}}^{\prime }}_{3}\right)\right]+2\sinh\left(2\beta {{\tilde{J}}^{\prime }}_{2}\right)\right\},\\ \; & {\tilde{\omega }}_{13}=2\cosh\left(\beta J_{1}^{\prime }\right)\left\{\sinh\left[2\beta \left({{\tilde{J}}^{\prime }}_{2}+{{\tilde{J}}^{\prime }}_{3}\right)\right]-\sinh\left[2\beta \left({{\tilde{J}}^{\prime }}_{2}-{{\tilde{J}}^{\prime }}_{3}\right)\right]+2\sinh\left(2\beta {{\tilde{J}}^{\prime }}_{3}\right)\right\},\\ \; & {\tilde{\omega }}_{14}=2\cosh\left(\beta J_{1}^{\prime }\right)\left\{\sinh\left[2\beta \left({{\tilde{J}}^{\prime }}_{2}+{{\tilde{J}}^{\prime }}_{3}\right)\right]-\sinh\left[2\beta \left({{\tilde{J}}^{\prime }}_{2}-{{\tilde{J}}^{\prime }}_{3}\right)\right]-2\sinh\left(2\beta {{\tilde{J}}^{\prime }}_{3}\right)\right\},\\ \; & {\tilde{\omega }}_{15}=-2\sinh\left(\beta J_{1}^{\prime }\right)\left\{\sinh\left[2\beta \left({{\tilde{J}}^{\prime }}_{2}+{{\tilde{J}}^{\prime }}_{3}\right)\right]-\sinh\left[2\beta \left({{\tilde{J}}^{\prime }}_{2}-{{\tilde{J}}^{\prime }}_{3}\right)\right]-2\sinh\left(2\beta {{\tilde{J}}^{\prime }}_{3}\right)\right\},\\ \; & {\tilde{\omega }}_{16}=-2\sinh\left(\beta J_{1}^{\prime }\right)\left\{\sinh\left[2\beta \left({{\tilde{J}}^{\prime }}_{2}+{{\tilde{J}}^{\prime }}_{3}\right)\right]-\sinh\left[2\beta \left({{\tilde{J}}^{\prime }}_{2}-{{\tilde{J}}^{\prime }}_{3}\right)\right]+2\sinh\left(2\beta {{\tilde{J}}^{\prime }}_{3}\right)\right\}. \end{array}$$

It can be examined that ${\tilde{\omega }}_{9}{\tilde{\omega }}_{10}={\tilde{\omega }}_{13}{\tilde{\omega }}_{14}$ and ${\tilde{\omega }}_{11}{\tilde{\omega }}_{12}={\tilde{\omega }}_{15}{\tilde{\omega }}_{16}$, such that the odd free-fermion condition Equation (12) is satisfied. Therefore, the triangular lattice Ising model in the imaginary field is equivalent with an odd free-fermion model. Now, the result is

(35) $$\begin{array}{cc} \; & \lim_{N\to \infty }\frac{1}{N}\ln Z=i\frac{\pi }{2}+\ln B+\ln A_{2}^{\prime }+\ln A_{3}^{\prime }+\lim_{N_{v}\to \infty }\frac{1}{N_{v}}\ln Z_{\mathrm{odd}}, \end{array}$$

where the exact solution of the odd free-fermion model is given by Equation (21) of Ref. [42]:

(36) $$\begin{array}{cc} \; & \lim_{N_{v}\to \infty }\frac{1}{N_{v}}\ln Z_{\mathrm{odd}}=\frac{1}{16\pi^{2}}\int_{0}^{2\pi }d\theta \int_{0}^{2\pi }d\phi \ln\left[a+b\cos\theta +c\cos\phi +d\cos\left(\theta -\phi \right)+e\cos\left(\theta +\phi \right)\right] \end{array}$$

with

(37) $$\begin{array}{cc} \; & a=2\left[{\left({\tilde{\omega }}_{9}{\tilde{\omega }}_{10}+{\tilde{\omega }}_{11}{\tilde{\omega }}_{12}\right)}^{2}+{\left({\tilde{\omega }}_{9}{\tilde{\omega }}_{12}\right)}^{2}+{\left({\tilde{\omega }}_{10}{\tilde{\omega }}_{11}\right)}^{2}+{\left({\tilde{\omega }}_{13}{\tilde{\omega }}_{16}\right)}^{2}+{\left({\tilde{\omega }}_{14}{\tilde{\omega }}_{15}\right)}^{2}\right],\\ \; & b=2\left[-{\left({\tilde{\omega }}_{9}{\tilde{\omega }}_{12}\right)}^{2}-{\left({\tilde{\omega }}_{10}{\tilde{\omega }}_{11}\right)}^{2}+2{\tilde{\omega }}_{13}{\tilde{\omega }}_{14}{\tilde{\omega }}_{15}{\tilde{\omega }}_{16}\right],\\ \; & c=2\left[{\left({\tilde{\omega }}_{13}{\tilde{\omega }}_{16}\right)}^{2}+{\left({\tilde{\omega }}_{14}{\tilde{\omega }}_{15}\right)}^{2}-2{\tilde{\omega }}_{9}{\tilde{\omega }}_{10}{\tilde{\omega }}_{11}{\tilde{\omega }}_{12}\right],\\ \; & d=-2{\left({\tilde{\omega }}_{9}{\tilde{\omega }}_{10}-{\tilde{\omega }}_{15}{\tilde{\omega }}_{16}\right)}^{2},\\ \; & e=-2{\left({\tilde{\omega }}_{11}{\tilde{\omega }}_{12}-{\tilde{\omega }}_{15}{\tilde{\omega }}_{16}\right)}^{2}. \end{array}$$

We should remark that the relation between our notation of weights and that of Ref. [42] is

$$\begin{array}{c} {\tilde{\omega }}_{9}=u_{1},\;{\tilde{\omega }}_{10}=u_{2},\;{\tilde{\omega }}_{11}=u_{4},\;{\tilde{\omega }}_{12}=u_{3},\;{\tilde{\omega }}_{13}=u_{7},\;{\tilde{\omega }}_{14}=u_{8},\;{\tilde{\omega }}_{15}=u_{6},\;{\tilde{\omega }}_{16}=u_{5}. \end{array}$$

Substituting $A_{i}^{\prime }=\frac{1}{2}e^{\beta J_{i}^{\prime }}$ and Equations (6), (7) and (34) into Equation (35), we have the final expression:

(38) $$\begin{array}{cc} \; & \lim_{N\to \infty }\frac{1}{N}\ln Z=i\frac{\pi }{2}+\frac{1}{16\pi^{2}}\int_{0}^{2\pi }d\theta \int_{0}^{2\pi }d\phi \ln\left[e^{-4\beta \left(J_{1}+J_{2}+J_{3}\right)}+e^{4\beta \left(J_{1}+J_{2}-J_{3}\right)}+e^{4\beta \left(J_{1}-J_{2}+J_{3}\right)}\right.\\ \; & \left.+e^{4\beta \left(-J_{1}+J_{2}+J_{3}\right)}-4-8\sinh^{2}\left(2\beta J_{3}\right)\cos\theta +8\sinh^{2}\left(2\beta J_{2}\right)\cos\phi -8\sinh^{2}\left(2\beta J_{1}\right)\cos\left(\theta -\phi \right)\right]. \end{array}$$

Then, we rederive the exact solution of this case [21,27] and indicate that the model in this case can be seen as an odd free-fermion model.

It is easy to find that this frustrated system (when $J_{1}=J_{2}=J_{3}>0$) in the imaginary field has a finite residual entropy at the zero-temperature limit:

(39) $$\begin{array}{c} S=\frac{1}{16\pi^{2}}\int_{0}^{2\pi }d\theta \int_{0}^{2\pi }d\phi \ln\left\{3-2\left[\cos\theta -\cos\phi +\cos\left(\theta -\phi \right)\right]\right\}=0.161533. \end{array}$$

Here, we set the Boltzmann constant $k_{B}=1$. Interestingly, the residual entropy in the imaginary field is half that in the zero field [32] (we can also obtain the residual entropy in the zero field with Equation (30) and compare the value with Equation (39)).

### 3.3. The Kagomé Lattice

**Figure 6:**
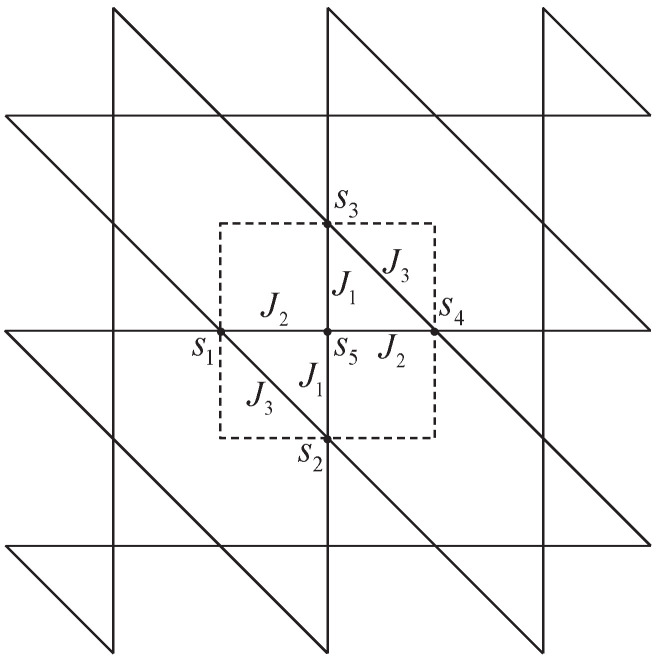
The diagrammatic representation of the Kagomé lattice. The region bounded by dash lines forms a vertex site of the sixteen-vertex model.

Figure 6 shows the Kagomé lattice. The region bounded by dash lines, which consists of two triangles, is chosen as the vertex site on the square lattice. The states of four spins $\left(s_{1},s_{2},s_{3},s_{4}\right)$ around each site can be mapped to the arrow configurations around this vertex by Equation (13); thus, the sixteen-vertex model is conveniently constructed.

#### 3.3.1. Zero-Field Case

The partition function is in the form of

(40) $$Z=\sum_{\left\{\left(s_{1},s_{2},s_{3},s_{4}\right)\right\}=\pm 1}\;\prod_{\mathrm{all}\;\mathrm{vertices}}\omega \left(s_{1},s_{2},s_{3},s_{4}\right)$$

with

(41) $$\begin{array}{cc} \; & \omega \left(s_{1},s_{2},s_{3},s_{4}\right)=\sum_{s_{5}=\pm 1}e^{-\beta J_{3}\left(s_{1}s_{2}+s_{3}s_{4}\right)}e^{-\beta \left[J_{1}s_{5}\left(s_{2}+s_{3}\right)+J_{2}s_{5}\left(s_{1}+s_{4}\right)\right]}. \end{array}$$

The translated vertex weights from the mapping in Equation (13) are

(42) $$\begin{array}{cc} \; & \omega_{1}=\omega_{2}=e^{-2\beta J_{3}}\left[e^{2\beta \left(J_{1}+J_{2}\right)}+e^{-2\beta \left(J_{1}+J_{2}\right)}\right],\\ \; & \omega_{3}=\omega_{4}=e^{2\beta J_{3}}\left[e^{2\beta \left(J_{1}-J_{2}\right)}+e^{-2\beta \left(J_{1}-J_{2}\right)}\right],\\ \; & \omega_{5}=\omega_{6}=2e^{2\beta J_{3}},\\ \; & \omega_{7}=\omega_{8}=2e^{-2\beta J_{3}},\\ \; & \omega_{9}=\omega_{10}=\omega_{11}=\omega_{12}=e^{2\beta J_{2}}+e^{-2\beta J_{2}},\\ \; & \omega_{13}=\omega_{14}=\omega_{15}=\omega_{16}=e^{2\beta J_{1}}+e^{-2\beta J_{1}}. \end{array}$$

Again, this symmetric sixteen-vertex model was transformed into an even eight-vertex model with Equation (8) with weights

(43) $$\begin{array}{c} {\tilde{\omega }}_{1}=e^{-2\beta J_{3}}\cosh\left[2\beta \left(J_{1}+J_{2}\right)\right]+e^{2\beta J_{3}}\cosh\left[2\beta \left(J_{1}-J_{2}\right)\right]+2\cosh\left(2\beta J_{3}\right)+2\cosh\left(2\beta J_{2}\right)+2\cosh\left(2\beta J_{1}\right),\\ {\tilde{\omega }}_{2}=e^{-2\beta J_{3}}\cosh\left[2\beta \left(J_{1}+J_{2}\right)\right]+e^{2\beta J_{3}}\cosh\left[2\beta \left(J_{1}-J_{2}\right)\right]+2\cosh\left(2\beta J_{3}\right)-2\cosh\left(2\beta J_{2}\right)-2\cosh\left(2\beta J_{1}\right),\\ {\tilde{\omega }}_{3}=e^{-2\beta J_{3}}\cosh\left[2\beta \left(J_{1}+J_{2}\right)\right]+e^{2\beta J_{3}}\cosh\left[2\beta \left(J_{1}-J_{2}\right)\right]-2\cosh\left(2\beta J_{3}\right)-2\cosh\left(2\beta J_{2}\right)+2\cosh\left(2\beta J_{1}\right),\\ {\tilde{\omega }}_{4}=e^{-2\beta J_{3}}\cosh\left[2\beta \left(J_{1}+J_{2}\right)\right]+e^{2\beta J_{3}}\cosh\left[2\beta \left(J_{1}-J_{2}\right)\right]-2\cosh\left(2\beta J_{3}\right)+2\cosh\left(2\beta J_{2}\right)-2\cosh\left(2\beta J_{1}\right),\\ {\tilde{\omega }}_{5}={\tilde{\omega }}_{6}=e^{-2\beta J_{3}}\cosh\left[2\beta \left(J_{1}+J_{2}\right)\right]-e^{2\beta J_{3}}\cosh\left[2\beta \left(J_{1}-J_{2}\right)\right]+2\sinh\left(2\beta J_{3}\right),\\ {\tilde{\omega }}_{7}={\tilde{\omega }}_{8}=e^{-2\beta J_{3}}\cosh\left[2\beta \left(J_{1}+J_{2}\right)\right]-e^{2\beta J_{3}}\cosh\left[2\beta \left(J_{1}-J_{2}\right)\right]-2\sinh\left(2\beta J_{3}\right). \end{array}$$

One can examine that ${\tilde{\omega }}_{1}{\tilde{\omega }}_{2}={\tilde{\omega }}_{7}{\tilde{\omega }}_{8}$ and ${\tilde{\omega }}_{3}{\tilde{\omega }}_{4}={\tilde{\omega }}_{5}{\tilde{\omega }}_{6}$; therefore, this case is an even free-fermion model. Making use of the even free-fermion solution as expressed in Equations (18) and (19), we give the exact result as follows:

(44) $$\begin{array}{c} \lim_{N\to \infty }\frac{1}{N}\ln Z\\ =\frac{1}{3}\lim_{N_{v}\to \infty }\frac{1}{N_{v}}\ln Z_{\mathrm{even}}\\ =\frac{1}{24\pi^{2}}\int_{0}^{2\pi }d\theta \int_{0}^{2\pi }d\phi \ln\left\{16\left[{\left(C_{1}C_{2}C_{3}-S_{1}S_{2}S_{3}\right)}^{2}+C_{1}^{2}+C_{2}^{2}+C_{3}^{2}\right]+32S_{1}\left(S_{1}C_{2}C_{3}-C_{1}S_{2}S_{3}\right)\cos\theta \right.\\ \;\;\;\;\;\;\;\;\;\;\;\;\;\;\;\;\;\;\;\;\;\;\;\;\;\;\;\;\;\;\;\;\;\left.+32S_{2}\left(C_{1}S_{2}C_{3}-S_{1}C_{2}S_{3}\right)\cos\phi -32S_{3}\left(C_{1}C_{2}S_{3}-S_{1}S_{2}C_{3}\right)\cos\left(\theta -\phi \right)\right\} \end{array}$$

with $C_{i}=\cosh\left(2\beta J_{i}\right)$ and $S_{i}=\sinh\left(2\beta J_{i}\right)$ (i=1,2,3). This result agrees with the known expressions (see Equation (36) of Ref. [37] for the isotropic case for an example).

#### 3.3.2. Imaginary Field Case

Taking into account Equation (22), the partition function in the presence of the imaginary field is

(45) $$Z=i^{N}\sum_{\left\{\left(s_{1},s_{2},s_{3},s_{4}\right)\right\}=\pm 1}\;\prod_{\mathrm{all}\;\mathrm{vertices}}\omega \left(s_{1},s_{2},s_{3},s_{4}\right)$$

with

(46) $$\begin{array}{cc} & \omega \left(s_{1},s_{2},s_{3},s_{4}\right)=\sum_{s_{5}=\pm 1}\left(s_{1}s_{2}s_{5}\right)e^{-\beta J_{3}\left(s_{1}s_{2}+s_{3}s_{4}\right)}e^{-\beta \left[J_{1}s_{5}\left(s_{2}+s_{3}\right)+J_{2}s_{5}\left(s_{1}+s_{4}\right)\right]}. \end{array}$$

We find that the resulting sixteen-vertex model is antisymmetric, like in the case of the triangular lattice model in the imaginary field. The vertex weights are listed as follows:

(47) $$\begin{array}{cc} \; & \omega_{1}=e^{-2\beta J_{3}}\left[e^{-2\beta \left(J_{1}+J_{2}\right)}-e^{2\beta \left(J_{1}+J_{2}\right)}\right],\\ \; & \omega_{2}=-\omega_{1},\\ \; & \omega_{3}=e^{2\beta J_{3}}\left[e^{-2\beta \left(J_{1}-J_{2}\right)}-e^{2\beta \left(J_{1}-J_{2}\right)}\right],\\ \; & \omega_{4}=-\omega_{3},\\ \; & \omega_{5}=\omega_{6}=\omega_{7}=\omega_{8}=0,\\ \; & \omega_{9}=\omega_{11}=e^{2\beta J_{2}}-e^{-2\beta J_{2}},\\ \; & \omega_{10}=\omega_{12}=-\omega_{9},\\ \; & \omega_{13}=\omega_{15}=e^{2\beta J_{1}}-e^{-2\beta J_{1}},\\ \; & \omega_{14}=\omega_{16}=-\omega_{13}. \end{array}$$

Then, the transformed weights of the odd eight-vertex model are

(48) $$\begin{array}{cc} \; & {\tilde{\omega }}_{9}=e^{-2\beta J_{3}}\sinh\left[2\beta \left(J_{1}+J_{2}\right)\right]-e^{2\beta J_{3}}\sinh\left[2\beta \left(J_{1}-J_{2}\right)\right]+2\sinh\left(2\beta J_{2}\right),\\ \; & {\tilde{\omega }}_{10}=e^{-2\beta J_{3}}\sinh\left[2\beta \left(J_{1}+J_{2}\right)\right]-e^{2\beta J_{3}}\sinh\left[2\beta \left(J_{1}-J_{2}\right)\right]-2\sinh\left(2\beta J_{2}\right),\\ \; & {\tilde{\omega }}_{11}={\tilde{\omega }}_{9},\\ \; & {\tilde{\omega }}_{12}={\tilde{\omega }}_{10},\\ \; & {\tilde{\omega }}_{13}=e^{-2\beta J_{3}}\sinh\left[2\beta \left(J_{1}+J_{2}\right)\right]+e^{2\beta J_{3}}\sinh\left[2\beta \left(J_{1}-J_{2}\right)\right]+2\sinh\left(2\beta J_{1}\right),\\ \; & {\tilde{\omega }}_{14}=e^{-2\beta J_{3}}\sinh\left[2\beta \left(J_{1}+J_{2}\right)\right]+e^{2\beta J_{3}}\sinh\left[2\beta \left(J_{1}-J_{2}\right)\right]-2\sinh\left(2\beta J_{1}\right),\\ \; & {\tilde{\omega }}_{15}={\tilde{\omega }}_{13},\\ \; & {\tilde{\omega }}_{16}={\tilde{\omega }}_{14}. \end{array}$$

It is straightforward to verify that ${\tilde{\omega }}_{9}{\tilde{\omega }}_{10}={\tilde{\omega }}_{13}{\tilde{\omega }}_{14}$ such that this case is equivalent with an odd free-fermion model; the exact result is thereby given by the odd free-fermion solution in Equations (36) and (37):

(49) $$\begin{array}{cc} \; & \;\;\;\;\lim_{N\to \infty }\frac{1}{N}\ln Z\\ \; & =i\frac{\pi }{2}+\frac{1}{3}\lim_{N_{v}\to \infty }\frac{1}{N_{v}}\ln Z_{\mathrm{odd}}\\ \; & =i\frac{\pi }{2}+\frac{1}{6}\ln\left[16\left|2S_{1}^{2}S_{2}^{2}S_{3}^{2}+S_{1}^{2}S_{2}^{2}+S_{1}^{2}S_{3}^{2}+S_{2}^{2}S_{3}^{2}-2S_{1}S_{2}S_{3}C_{1}C_{2}C_{3}\right|\right] \end{array}$$

with $C_{i}=\cosh\left(2\beta J_{i}\right)$ and $S_{i}=\sinh\left(2\beta J_{i}\right)$ (i=1,2,3). The expression is surprisingly simple. To our knowledge, this result has not been reported previously. There has research studying the Kagomé lattice Ising model in a magnetic field through transformation into the honeycomb lattice model [89,90,91]. One can examine that the case in the imaginary field was not included in such works. Our analysis demonstrates that this case is also exactly solvable.

We note that the details of the mapping and calculation procedure are very important. In this case, the vertex weights are defined in Equation (46), in which the factor $\prod_{i=1}^{N}s_{i}$ in the partition function exhibits a subfactor $s_{1}s_{2}s_{5}$. One can see that the effect of the field is partitioned as $e^{\beta H_{\mathrm{ex}}\left(s_{1}+s_{2}+s_{5}\right)}$ for this vertex unit. Now, we repartition this effect as $e^{\beta H_{\mathrm{ex}}\left[\frac{1}{2}\left(s_{1}+s_{2}+s_{3}+s_{4}\right)+s_{5}\right]}$, i.e., the vertex weights are reset as

$$\begin{array}{c} \omega \left(s_{1},s_{2},s_{3},s_{4}\right)=\sum_{s_{5}=\pm 1}\left(s_{1}^{1/2}s_{2}^{1/2}s_{3}^{1/2}s_{4}^{1/2}s_{5}\right)e^{-\beta J_{3}\left(s_{1}s_{2}+s_{3}s_{4}\right)}e^{-\beta \left[J_{1}s_{5}\left(s_{2}+s_{3}\right)+J_{2}s_{5}\left(s_{1}+s_{4}\right)\right]} \end{array}$$

with $1^{1/2}=1$ and ${\left(-1\right)}^{1/2}=i$. In this way, we will find that

$$\begin{array}{cc} \; & \omega_{1}=-\omega_{2},\;\omega_{3}=-\omega_{4},\;\omega_{5}=\omega_{6}=\omega_{7}=\omega_{8}=0,\\ \; & \omega_{9}=\omega_{10},\;\omega_{11}=\omega_{12},\;\omega_{13}=\omega_{14},\;\omega_{15}=\omega_{16}. \end{array}$$

We obtain neither a symmetric nor an antisymmetric sixteen-vertex model. Hence, we fail to map the Ising model into an even or odd free-fermion model by weak-graph expansion. We will probably miss this new solvable case, or at least we are not able to solve this case so conveniently by mapping onto the odd free-fermion model as we have shown. This fact indicates that the details of mapping and calculation may play a key role in obtaining the new solution.

As is well known, most of the exact solutions of two-dimensional Ising models are of the form of a double integral. Onsager’s solution of the square lattice model [5] is a very early example. Each model studied in this paper using the free-fermion approach involves a double integral in the solution, except for the Kagomé lattice model in the imaginary field. It is interesting to compare the result of Equation (49) with Equation (1) of Ref. [92], which is the solution of the dimer model on the Kagomé lattice and can also be derived by mapping to the odd free-fermion model. Both formulas take a very simple logarithmic form. Ref. [92] stated that the novel and unique expression “points to the special role played by the Kagomé lattice”. Here, we provide new evidence for this statement.

In the isotropic case that $J_{1}=J_{2}=J_{3}=J$, Equation (49) reduces to

(50) $$\begin{array}{c} \lim_{N\to \infty }\frac{1}{N}\ln Z=i\frac{\pi }{2}+\frac{1}{6}\ln\left[\left(1+3e^{4\beta J}\right){\left|1-e^{-4\beta J}\right|}^{3}\right]. \end{array}$$

It is straightforward to verify that, this case does not exhibit a physical phase transition. When J>0, the Kagomé lattice Ising model is geometrically frustrated. The residual entropy in the imaginary field is easily calculated from Equation (50) by taking the zero temperature limit:

(51) $$S=\frac{1}{6}\ln3=0.183102.$$

## 4. Summary

The exact solutions of Ising models on typical two-dimensional lattices, specifically the honeycomb, triangular, and Kagomé lattices, are studied in the free-fermion formulation. For each Ising model, both the case of a zero field and the case of an imaginary field i(ππ22)kBT are considered. Five known solutions are rederived, and one new solution is found. In particular, the Kagomé lattice model in the imaginary field is shown to be exactly solvable, exhibiting a very simple form of solution. Hence, we introduced a new member to the family of exactly solvable lattice models.

All solutions in this paper are achieved by mapping the Ising spin system into a free-fermion model. The mapping procedure is straightforward and should be easy to extend to other lattice systems. Applying the mapping method to the Ising model in a physical (real) and non-zero magnetic field would be interesting. Furthermore, the free-fermion formulation may be suitable for other statistical lattice problems, e.g., the dimer covering problem. Our work has provided new insights into the mathematically exact results of spin systems, showcasing the elegance and power of the free-fermion formulation in condensed matter and statistical physics.

## Data Availability

No new data were created or analyzed in this study. Data sharing is not applicable to this article.
